# Deep sequencing analysis of tick-borne encephalitis virus from questing ticks at natural foci reveals similarities between quasispecies pools of the virus

**DOI:** 10.1099/jgv.0.000704

**Published:** 2017-04-01

**Authors:** Naveed Asghar, John H.-O Pettersson, Patrik Dinnetz, Åshild Andreassen, Magnus Johansson

**Affiliations:** ^1^​ School of Natural Science, Technology & Environmental Studies, Södertörn University, Huddinge, Sweden; ^2^​ School of Medical Sciences, Örebro University, Örebro, Sweden; ^3^​ iRiSC – Inflammatory Response and Infection Susceptibility Centre, Faculty of Medicine and Health, Örebro University, Örebro, Sweden; ^4^​ Department of Infectious Disease Epidemiology and Modelling, Norwegian Institute of Public Health, Oslo, Norway; ^5^​ Department of Microbiology, National Veterinary Institute, Uppsala, Sweden; ^6^​ Department of Medical Biochemistry and Microbiology (IMBIM), Zoonosis Science Center, Uppsala University, Uppsala, Sweden; ^7^​ Department of Virology, Division of Infectious Disease Control, Norwegian Institute of Public Health, Oslo, Norway

**Keywords:** *Ixodes ricinus*, natural foci, non-coding region, quasispecies, Scandinavia, tick-borne encephalitis virus

## Abstract

Every year, tick-borne encephalitis virus (TBEV) causes severe central nervous system infection in 10 000 to 15 000 people in Europe and Asia. TBEV is maintained in the environment by an enzootic cycle that requires a tick vector and a vertebrate host, and the adaptation of TBEV to vertebrate and invertebrate environments is essential for TBEV persistence in nature. This adaptation is facilitated by the error-prone nature of the virus’s RNA-dependent RNA polymerase, which generates genetically distinct virus variants called quasispecies. TBEV shows a focal geographical distribution pattern where each focus represents a TBEV hotspot. Here, we sequenced and characterized two TBEV genomes, JP-296 and JP-554, from questing *Ixodes ricinus* ticks at a TBEV focus in central Sweden. Phylogenetic analysis showed geographical clustering among the newly sequenced strains and three previously sequenced Scandinavian strains, Toro-2003, Saringe-2009 and Mandal-2009, which originated from the same ancestor. Among these five Scandinavian TBEV strains, only Mandal-2009 showed a large deletion within the 3′ non-coding region (NCR), similar to the highly virulent TBEV strain Hypr. Deep sequencing of JP-296, JP-554 and Mandal-2009 revealed significantly high quasispecies diversity for JP-296 and JP-554, with intact 3′NCRs, compared to the low diversity in Mandal-2009, with a truncated 3′NCR. Single-nucleotide polymorphism analysis showed that 40 % of the single-nucleotide polymorphisms were common between quasispecies populations of JP-296 and JP-554, indicating a putative mechanism for how TBEV persists and is maintained within its natural foci.

## Introduction

Tick-borne encephalitis (TBE) is a severe central nervous system infection, affecting between 10 000 and 15 000 people in Europe and Asia annually [1]. The causative agent, TBE virus (TBEV), is transmitted to humans through tick bites. TBEV is divided into three subtypes: European- (Eu-TBEV), Siberian and Far Eastern [2, 3]. The names correspond to the principal geographical distribution of these subtypes. TBE is known for its focal geographical distribution pattern where each focus represents a TBEV hotspot [4, 5]. TBE is endemic in central and southern Sweden, with the Stockholm archipelago representing the area with the highest number of TBEV foci [6].

TBEV is a single-stranded positive-sense RNA virus that comprises a single ORF flanked by 5′ and 3′ non-coding regions (NCRs). The 3′NCR consists of a conserved fragment (C 3′NCR) comprising about 340–350 terminal nucleotides and an upstream variable fragment (V 3′NCR) [7, 8]. In 2003, a new TBEV focus was identified at Torö (58° 49′ N 17° 50′ E) in the south of Stockholm, and Toro-2003 represents the first TBEV strain from Torö [9]. In 2008, five years after the identification of Toro-2003, we sampled additional questing ticks at Torö and amplified two new TBEV genomes, JP-296 and JP-554, from virus-positive *Ixodes ricinus* ticks. The new strains were phylogenetically characterized as Eu-TBEV. Because V 3′NCR is the least stable region within the Eu-TBEV genome, we evaluated the stability and evolution of Eu-TBEV within its natural focus by comparing the 3′NCR organization of JP-296 and JP-554 with that of Toro-2003.

Compared to other viruses, arboviruses like TBEV are genetically constrained by selection, while they replicate in ectothermic invertebrate vectors and endothermic vertebrate hosts. Successful viral replication requires adaptation to both environments without loss of functional integrity. The error-prone nature of the RNA-dependent RNA polymerase of arboviruses is known to generate approximately 1 error per 10^4^ nucleotides per viral replication cycle, thus generating diverse virus populations that undergo selection to produce genetically distinct variants called quasispecies [10, 11]. The diversity of the quasispecies facilitates virus survival and adaptation in diverse environments and is critical for its evolution [12].

The zoonotic life cycle of TBEV involves ticks and vertebrate hosts [13, 14]. Previous studies have demonstrated that the existence of quasispecies within infected hosts is a putative virulence factor of TBEV. However, the quasispecies dynamics and evolution in nature is less well understood [15–17]. Such knowledge would be important for understanding the virulence diversity of TBEV and might have a role in the development of attenuated live vaccine candidates. Here, we performed deep sequencing on TBEV strains that were directly amplified from the questing ticks. Interestingly, the deep sequencing analysis revealed novel similarity regarding the population diversity of closely related viruses isolated from a natural local focus.

## Results

### Sequencing of TBEV genomes

With the increasing prevalence and incidence of TBE, there is a constant demand for new TBEV genomic sequences to improve our current understanding of the evolution and phylogeographic distribution of TBEV. Here, we generated two TBEV genomic sequences, JP-296 and JP-554, from ticks sampled at an established TBEV focus in Torö, Sweden. Both genomes were amplified into seven overlapping DNA fragments using an established nested PCR technique. The overlapping PCR fragments were conventionally sequenced to generate consensus genomes of JP-296 and JP-554.

### Molecular evolution of TBEV

**Fig. 1.**
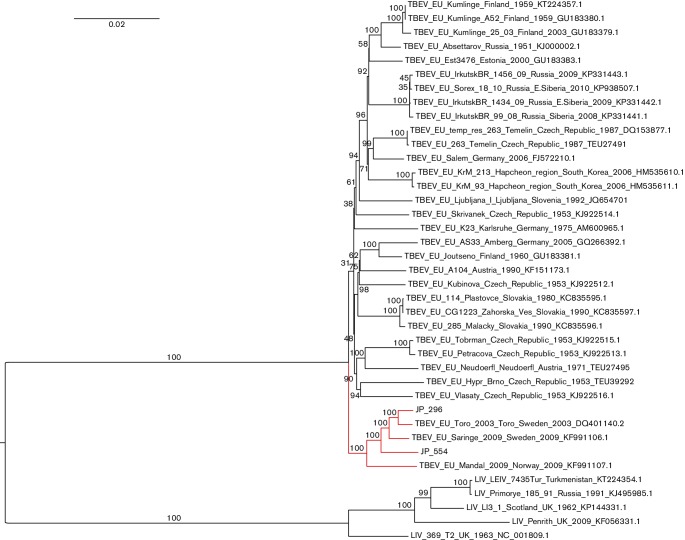
Phylogenetic analysis of the complete ORFs of JP-296 and JP-554, including 32 additional Eu-TBEV genomes and 5 LIV genomes, using the maximum-likelihood approach. The tree was inferred from 1000 rapid bootstrap replicates using the GTR + gamma model of molecular evolution. The Scandinavian clade is indicated in red.

The phylogenetic positions of JP-296 and JP-554 were inferred based on complete genomes and partial E genes using the maximum-likelihood approach. In both analyses, TBEV was rooted with louping ill virus (LIV), and the new strains were both classified as Eu-TBEV. In the complete genome phylogeny, our sequences form a well-supported Scandinavian clade that is basal to all other Eu-TBEV strains (bootstrap support=100 %) (Fig. 1). Notably, the Swedish TBEV strains JP-296, Saringe-2009 and JP-554 were more closely related to Toro-2003 than Mandal-2009, thus supporting the closer geographical relationship among the Eu-TBEV strains detected in ticks sampled from the Stockholm archipelago. It is important to note that although they were only collected 3 weeks apart from the same location, JP-296 and JP-554 appear to have originated from two different sources. The same phylogeographic clustering was also indicated in the partial E gene phylogeny, although it was less supported compared to the complete genome phylogeny (Fig. S1, available in the online Supplementary Material).

### 3′NCR genomic organization of Scandinavian TBEV strains

**Fig. 2.**
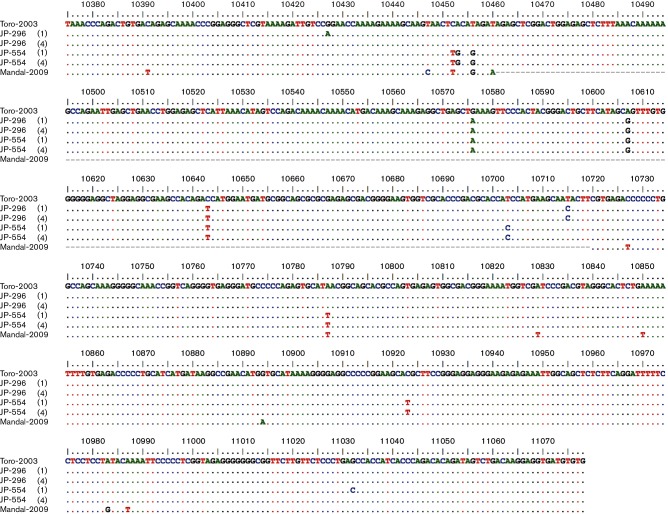
Alignment of the 3′NCR sequences of JP-296 and JP-554, obtained from respective pcDNA3.1 clones, with other Scandinavian TBEV strains sequenced directly from *I.* *ricinus*. Nucleotide numbers correspond to the TBEV strain Toro-2003 (GenBank accession no. DQ401140.2). Numbers indicating JP-296 and JP-554 clones with identical sequences are shown in parentheses.

Despite high genomic conservation, the V 3′NCR of Eu-TBEV is highly variable. We investigated the 3′NCR of the Scandinavian TBEV strains to identify variations among the strains, and the alignment of the 3′NCR sequences of JP-296 and JP-554 with other Scandinavian TBEV strains sequenced from ticks showed visible differences (Fig. 2). The 3′NCRs of JP-296 and JP-554 were identical in length to Toro-2003, whereas Saringe-2009 and Mandal-2009 contained the longest and shortest 3′NCRs, respectively. One out of the five sequenced clones of both JP-296 and JP-554 differed from the rest by a single nucleotide, indicating the presence of more than one TBEV variant in these samples.

### Quasispecies populations of TBEV strains with different 3′NCR organization

Because the 3′NCRs of the new Torö strains JP-296 and JP-554 were substantially different from the TBEV strain Mandal-2009, we decided to perform deep sequencing to explore the differences in the quasispecies populations of these strains. Single-nucleotide polymorphism (SNP) analysis was performed to identify and compare the population structures of TBEV strains with intact and truncated 3′NCRs. The deep sequencing results were aligned against the consensus sequence of each genome, and SNPs were calculated as a percentage. The SNP analysis showed that quasispecies exist within the samples of the studied viruses. The TBEV strain Mandal-2009 with a truncated V 3′NCR had a smaller number of SNPs compared to JP-296 and JP-554, both of which contain intact V 3′NCRs (Table 1). There were 16 SNPs that were common between JP-296 and JP-554, and these made up 40 and 41 % of the total SNPs in these strains, respectively. Out of the 16 SNPs shared by JP-296 and JP-554, 2 SNPs resulted in amino acid changes and 14 were silent mutations (Table 1). About 94 % of the common SNPs were localized within the part of the genome encoding for structural proteins. Taken together, our data show the similarity between the TBEV quasispecies pools of two questing ticks sampled from a natural TBEV focus.

**Table 1.** SNPs (>2 %) calculated for JP-296, JP-554 and Mandal-2009 compared to their respective consensus genomes SNPs in common between the JP-296 and JP-554 strains are underlined. A.A, amino acid; C, capsid gene; prM, precursor membrane gene; E, envelope gene; NS non-structural genes; NCR, non-coding region.

Position∗	Mutation	Region	A.A change	Depth (**%** SNP)		
				Mandal-2009	JP-296	JP-554
61	T to C	5′NCR	None			274 (3)
153	A to G	C	None	334 (3)		
259	T to C	C	None		1333 (21)	1170 (17)
282	T to C	C	None			1143 (13)
353	A to G	C	Lys to Arg			1263 (4)
370	G to A	C	Gly to Ser		1469 (13)	
372	T to C	C	None			1354 (11)
388	C to T	C	None		1414 (10)	
448	A to G	C	Thr to Ala		1549 (11)	1442 (13)
500	C to A	prM	Ser to†			1418 (2)
528	T to C	prM	None		1532 (14)	1309 (4)
546	C to T	prM	None			1335 (3)
570	T to C	prM	None			1428 (4)
575	T to C	prM	Val to Ala		1645 (14)	
654	C to T	prM	None		1546 (11)	1398 (6)
663	T to C	prM	None			1378 (5)
716	G to A	prM	Arg to Gln		1529 (13)	
723	C to G	prM	None		1521 (12)	1543 (9)
738	C to T	prM	None			1527 (5)
933	G to T	prM	None			1531 (13)
957	C to T	prM	None		1308 (16)	1602 (13)
984	A to G	E	None		1158 (6)	1552 (6)
993	T to C	E	None		1189 (23)	1636 (26)
999	T to G	E	None		1317 (23)	1745 (30)
999	T to C	E	None		1317 (12)	1745 (12)
1211	C to T	E	Ala to Val	366 (3)		
1297	A to G	E	Ser to Gly	655 (4)		
1432	C to T	E	His to Tyr		4259 (2)	2026 (2)
1475	A to C	E	Lys to Thr	734 (3)		
1595	C to T	E	Thr to Met		2265 (2)	
1941	A to G	E	None	750 (2)		
2277	T to C	E	None		1825 (49)	
2277	C to T	E	None			1129 (41)
2283	T to C	E	None		1542 (33)	1022 (34)
2286	T to C	E	None		1518 (17)	1020 (19)
2292	T to C	E	None			715 (3)
2295	C to A	E	None		886 (24)	775 (25)
2695	G to T	NS1	Gly to Trp		2498 (2)	
2837	A to G	NS1	Glu to Gly			2127 (4)
3039	A to G	NS1	None			2172 (2)
3066	G to T	NS1	Met to Ile		2829 (2)	
3276	A to G	NS1	None		2614 (5)	
3349	A to G	NS1	Arg to Gly		2341 (2)	
3743	T to C	NS2A	Met to Thr			927 (31)
4104	C to T	NS2A	None		1318 (3)	
4192	A to G	NS2A	Arg to Gly			1011 (11)
4199	C to T	NS2B	Ser to Phe		1212 (2)	
4539	T to A	NS2B	None			1049 (2)
4542	T to A	NS2B	None		1317 (25)	
4542	A to T	NS2B	None			1016 (52)
5192	C to T	NS3	Pro to Leu			1768 (11)
5236	C to T	NS3	Gln to†		2587 (4)	
5452	A to G	NS3	Ile to Val			2110 (7)
5531	A to G	NS3	Lys to Arg		2514 (8)	
5608	A to G	NS3	Ser to Gly			1296 (3)
5642	G to A	NS3	Arg to His		983 (13)	
5984	G to T	NS3	Gly to Val	949 (2)		
6014	T to C	NS3	Ile to Thr			1272 (5)
6399	G to A	NS3	Met to Ile		1024 (14)	
6726	C to G	NS4A	Phe to Leu		954 (77)	
7191	C to T	NS4B	None			855 (22)
7737	C to T	NS5	None			1055 (2)
7738	A to G	NS5	Arg to Gly			1047 (27)
7761	G to A	NS5	None	1059 (2)		
7858	C to A	NS5	Arg to Ser		680 (3)	
7883	A to G	NS5	Glu to Gly	405 (3)		
7936	C to T	NS5	Arg to†		591 (2)	
7972	G to A	NS5	Gly to Ser		852 (17)	
8388	G to T	NS5	None		1242 (3)	
9346	C to T	NS5	Gln to†		1558 (4)	
9839	C to T	NS5	Pro to Leu		115 (5)	
9852	A to G	NS5	None		113 (2)	
11 040	A to G	3′NCR	None			378 (35)

*The positions correspond to JP-296 GenBank accession no. KX966398.

†Stop codon.

### Mutational profiles of TBEV strains with different 3′NCRs

**Fig. 3.**
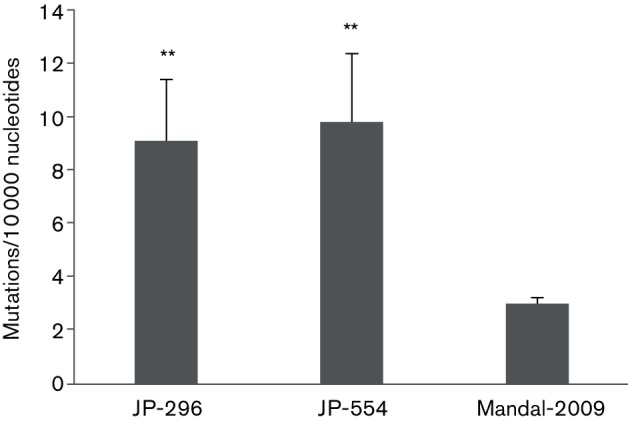
Average number of mutations per 10 000 nucleotides calculated for the two Torö strains and Mandal-2009. Error bars correspond to the standard error of the mean. Asterisks denote a significant difference compared to Mandal-2009 (*P*<0.01, Tukey's post hoc test).

To analyse population diversity and to identify the genes responsible for such diversity, mutation frequencies were calculated for JP-296, JP-554 and Mandal-2009. The population diversity of the new Torö strains containing intact V 3′NCRs was significantly higher than that of Mandal-2009 (Fig. 3). The observed variance was primarily due to differences in the mutation frequencies of structural genes. Mandal-2009 showed fairly constant mutation frequencies over the whole ORF, whereas JP-296 and JP-554 showed noticeable differences in the mutation frequencies of individual genes (data not shown).

## Discussion

In Scandinavia, new natural TBEV foci are constantly emerging [6, 18], which has led to an increase in the prevalence and incidence of TBE in recent decades [19, 20]. Many factors contribute to the clinical outcome of TBE, which varies from mild and asymptomatic to severe and fatal. The strain-specific virulence underlines the importance of identifying and genetically characterizing specific TBEV strains from novel foci. In this study, we sequenced and characterized two new TBEV strains, JP-296 and JP-554, from a natural TBEV focus in Torö in southern Stockholm. The two new Torö strains were phylogenetically grouped with Toro-2003, the first TBEV strain isolated from the same area. All five Eu-TBEV strains that formed a Scandinavian clade in the phylogenetic analysis (Fig. 1) represent TBEV genomes that were amplified directly from *I. ricinus* without being cultivated in vertebrate cells. JP-296 and JP-554 were amplified from individual questing adult male ticks, Toro-2003 was amplified from a pool of questing ticks (9 adults and 106 nymphs) [9], Mandal-2009 was amplified from a pool of 10 questing nymphs [15] and Saringe-2009 was amplified from a single blood-fed female tick [15].

In the study by Weidmann *et al.* [21], two Eu-TBEV lineages (A and B) were identified in central Europe. In the study, six TBEV foci with multiple E gene sequences (*n*≥5) were used to characterize local genetic diversity within TBEV foci during a few decades [21]. The phylogenetic analyses based on the complete genome and partial E gene (Figs 1 and S1) includes strains of the major central- and eastern European Eu-TBEV lineages and showed a distinct clade, dominated by Scandinavian strains separate from all other Eu-TBEV clades.

TBEV quasispecies are known to exist as populations of mixed variants both in ticks and mammals [16, 17]. The switching of hosts results in the selection of best-fit variants, but the less-fit variants keep replicating at lower levels in the new host and can re-emerge after switching back to the original host [16]. However, studies exploring TBEV quasispecies structure within the natural life cycle of TBEV are missing. Virus cultivation in cell culture or mice can alter its natural quasispecies structure. For example, changes in the genetic composition of bovine herpes virus 1, West Nile virus and TBEV populations have all been observed after a single passage in their respective hosts [16, 22, 23]. Contrary to most of the available TBEV sequences, our sequences were generated directly from questing *I. ricinus* without laboratory cultivations, thus making them ideal candidates to study the population structure of viral quasispecies in nature. Deep sequencing of JP-296, JP-554 and Mandal-2009 revealed TBEV quasispecies structure within *I. ricinus*. We were unable to perform deep sequencing for Toro-2003 because the RNA from the Toro-2003 pool of questing ticks had been consumed in previous studies.

It has recently been demonstrated that the structural genes of Eu-TBEV are prime determinants for TBEV replication and non-viremic transmission among co-feeding *I. ricinus*, whereas the non-structural genes regulate the cytopathogenicity of TBEV [24]. The genes encoding the structural proteins of JP-296, JP-554 and Mandal-2009 exhibited the highest numbers of SNPs (data not shown), whereas in another study, the NS2A gene of cDNA-derived TBEV exhibited the highest number of SNPs after cultivation in cell culture and in mice [8, 25, 26]. Together, these findings suggest that certain viral genes behave differentially in vertebrate and invertebrate cells. Most of the SNPs that were common between JP-296 and JP-554 represent synonymous mutations (Table 1). The structural proteins are critical for the survival, transmission and replication of TBEV in invertebrate and vertebrate environments, and the synonymous mutations represent strict selection constraints on amino acid changes faced by TBEV within the tick vector. Similar selective constraints on amino acid changes of the capsid proteins of vector-borne plant viruses have also been reported [27]. In addition, Chare and Holmes [27] showed that the purifying selection was greater at the virus–vector interface than the virus–host interface. The increased codon diversity due to these synonymous mutations might allow for different levels of gene expression that might provide an advantage in different hosts.

The V 3′NCR is highly heterogeneous, both in its length and its nucleotide sequence, among the TBEV strains and is an important virulence determinant of the virus [8, 25, 26]. Deletions within the V 3′NCR have been reported following virus cultivation in cell culture or suckling mouse brains, whereas the complete 3′NCR was proposed to be necessary for efficient virus replication in ticks [28]. In addition, TBEV strains with deletions in V 3′NCR have been isolated from severe TBE patients [29]. However, TBEVs with truncated V 3′NCR do exist in nature [15], as exemplified by the Mandal-2009 strain. The uniformity in the V 3′NCR genomic organization of the other three Scandinavian strains Toro-2003, JP-296 and JP-554 indicates the stability of Eu-TBEV within a natural focus. In addition, the 40 % SNP identity in the population structures of the two new Torö strains indicates the stability of virus quasispecies pools within infected ticks sampled at a TBEV focus. Interestingly, the quasispecies diversity of Mandal-2009, which contains a truncated V 3′NCR, was significantly lower than that of the two Torö strains with intact V 3′NCRs. The relatively poor quasispecies diversity observed in the case of Mandal-2009 could be due to the higher genomic stability of shorter TBEV variants or the low virus titre of the sample [30]. After infection, ticks become persistent carriers of TBEV for the rest of their lives [31, 32]. The low virus titres in Mandal-2009 might be due to a dilution effect because of pooling (*n*=10 nymphs), a slower replication rate, or having spent less time in the tick after infection – assuming that all three TBEV-positive tick samples were infected at the same stage of the tick life cycle. Additional evidence for the difference in quasispecies due to the time spent in the tick is the observation of a 100-fold increase in Powassan virus titres during the larvae-to-nymph moulting of deer ticks [33].

In summary, we have extensively characterized novel TBEV strains, demonstrating a phylogeographical clustering among five Scandinavian strains. Deep sequencing of three of the strains revealed quasispecies diversity, possibly related to the genomic organization of TBEVs with long and short 3′NCRs, respectively. However, SNP analysis demonstrated 40 % similarity between the quasispecies populations of strains with long 3′NCRs identified at nearby foci, which indicates a putative mechanism for the persistence of quasispecies populations of closely related TBEV strains.

## Methods

### TBEV strains

The three TBEV strains investigated in this study were JP-296, JP-554 and Mandal-2009. The first two strains were sequenced from male *I. ricinus* sampled near Torö, Sweden (58° 48.42′ N 17° 49.56′ E) [6], and Mandal-2009 came from a TBEV-positive pool of 10 questing nymphs sampled at Mandal, Norway (58° 0.43′ N 7° 30.00′ E) [34]. Real-time PCR analysis on the primary RNA of Mandal-2009, JP-296 and JP-554 showed *C*_t_ values of 24.0, 16.5 and 17.2, respectively.

### RNA extraction and cDNA synthesis

Total RNA was extracted from homogenized ticks as described previously [15]. cDNA was synthesized using pd(N)6 random hexamer primers or the TBEV 3′NCR specific reverse primer (5′-GGGTGTTTTTCCGAGTCAC-3′) and Superscript III reverse transcriptase (Invitrogen) as per the manufacturer’s instructions. RNase H was used to digest the parental RNA.

### Nested PCR and sequencing

Nested PCR was performed using KOD Hot Start Master Mix (Novagen) to generate seven overlapping fragments covering the whole genome. Primers and reaction conditions for the nested PCR have been described elsewhere [15]. The consensus sequence of the Mandal-2009 genome is already available from the NCBI GenBank (KF991107). To obtain complete genomic sequences of JP-296 and JP-554, PCR products were gel extracted using the Wizard SV Gel and PCR Clean-Up System (Promega) and sent for sequencing (Eurofins MWG Operon). Genomic sequences of JP-296 and JP-554 were deposited in the NCBI GenBank (KX966398 and KX966399, respectively).

### Phylogenetic analysis

To analyse the phylogenetic relationship of JP-296 and JP-554, two alignments were constructed using Mafft [35]. The first was a complete genome alignment (10 245 bp), including 34 complete Eu-TBEV genomes and 5 louping ill virus (LIV) genomes. The second was a partial envelope (E) gene alignment (757–1480 bp), including 212 unique partial TBEV E gene sequences and 15 partial LIV E gene sequences. Maximum-likelihood trees were constructed for the complete genome and partial E gene alignments using RAxML [36] with 1000 rapid bootstrap replicates and the GTR + gamma model of molecular evolution. The resulting trees were viewed and annotated in FigTree (http://tree.bio.ed.ac.uk/software/figtree/). All computations were performed at the CIPRES Science Gateway [37].

### 3′ NCR analysis

The 3′NCRs of JP-296 and JP-554 were cloned into pcDNA3.1/V5-His-TOPO (Invitrogen) as per the manufacturer’s instructions, and five random clones of each strain were sequenced (Eurofins MWG Operon). The 3′NCR sequences of JP-296 and JP-554 were aligned against the 3′NCRs of other Scandinavian TBEV strains, which were sequenced directly from ticks without culturing in the laboratory. BioEdit (version 7.1.3.0; Tom Hall Ibis Therapeutics) was used to analyse the nucleotide sequences.

### Next-generation sequencing

For each strain, the seven overlapping PCR fragments were gel purified using the Wizard SV Gel and PCR Clean-Up System (Promega) and pooled in a 1 : 1 molar ratio. The concentration of each pool was quantified with the Qubit dsDNA BR Assay Kit (Life Technologies), and a final concentration of 0.15 ng µl^−1^ was achieved by diluting with 10 mM Tris pH 8.5. Indexed paired-end multiplexed sequencing libraries were prepared using the Nextera XT DNA library preparation kit (Illumina). The tagmentation reaction was performed at 55 °C for 6.5 min using 0.75 ng input DNA. Standard index primers (Illumina) were used for PCR amplification followed by purification with Agencourt AMPure XP beads (Beckman Coulter) as per the manufacturer’s instructions. The concentration of each library was measured with the Qubit dsDNA BR Assay Kit, and the size distribution was determined with the Agilent High Sensitivity DNA kit (Agilent Technologies). The normality of each library was calculated using the formula *χ*=(DNA concentration×10^6^)/(656.6×average size of fragments). Libraries were normalized, pooled and denatured using 0.2 M NaOH. The final denatured libraries were transferred to the MiSeq cartridge v2, 300 cycles (Illumina). The cartridge was loaded into the Miseq desktop sequencer (Illumina), and sequencing was performed using index reads 2 and paired-end settings.

### Deep sequencing data analysis

To assess the quality of the next-generation sequencing (NGS) reads, QC statistics were generated with FastQC (www.bioinformatics.bbsrc.ac.uk/projects/fastqc). The reads were trimmed and sorted using an average quality score of >30. For JP-296 and JP-554, the reads were aligned to the respective consensus TBEV genomic sequences generated by Sanger sequencing. For Mandal-2009, the NGS reads were aligned against the complete genomic sequence of Mandal-2009 (GenBank accession no. KF991107) using TopHat2 [38]. To calculate the occurrence of SNP, PCR duplicates were removed (Picard tools, http://broadinstitute.github.io/picard/) and files were converted (SAMtools [39]) to generate base counts with the UnifiedGenotyper from the GATK package [40].

### Mutation frequency analysis

Mutation frequencies were calculated for all of the TBEV genes by dividing the sum of mutations within a given gene by the total number of nucleotides sequenced for the respective gene. Mutation frequencies were presented per 10 000 nucleotides. Differences in mutation frequencies among the TBEV strains were analysed using a general linear mixed model with mutation frequency as a function of the fixed variable *strain* and the random variable *gene*. In this way, we used gene as a random replicate of strain.

### Statistical analysis

All analyses were carried out with the statistical package R 3.0.1 [41] and the additional packages lme4 [42], effects [43] and multcomp [44].

## Supplementary Data

74 Supplementary File 1
