# Supplementary material for: Deep sequencing analysis of tick-borne encephalitis virus from questing ticks at natural foci reveals similarities between quasispecies pools of the virus
Source: J Gen Virol. 2017 Apr 1;98(3):413–21. doi: 10.1099/jgv.0.000704 (PMC5797951; doi:10.1099/jgv.0.000704)

Figure S1.

Maximum likelihood phylogenetic analysis based on partial E gene alignment (757–1480bp), using 212 unique TBEV and 15 LIV partial E gene sequences. The Swedish strains within lineage C are indicated in red.

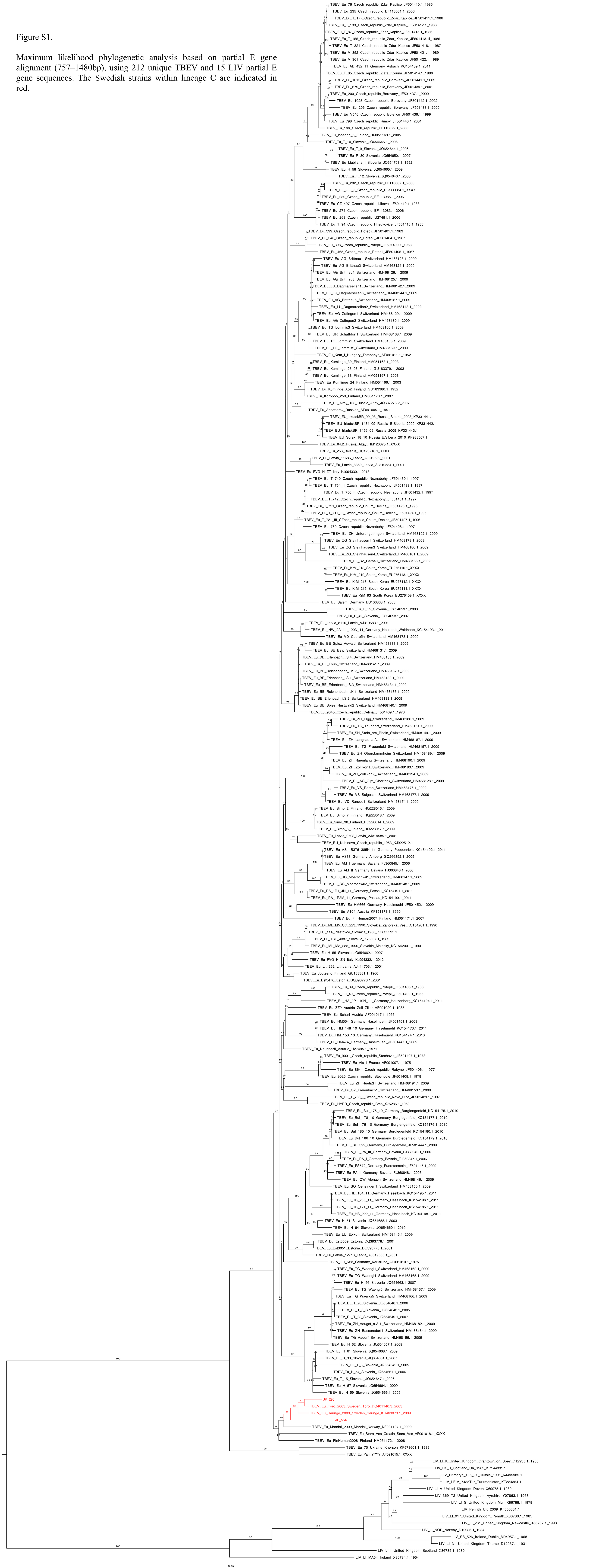

Supplement: Supplementary File 1 [file jgv-98-413-s001.pdf]
